# Body composition and rupture risk of intracranial aneurysms

**DOI:** 10.1186/s40001-024-01888-3

**Published:** 2024-05-24

**Authors:** Katja Løvik, Johnny Laupsa-Borge, Nicola Logallo, Christian A. Helland

**Affiliations:** 1 Department of Clinical Medicine, University of Bergen, Bergen, Norway; 2 Department of Clinical Science, University of Bergen, Bergen, Norway; 3 Department of Neurosurgery, Haukeland University Hospital, Bergen, Norway

**Keywords:** Body mass index, Cardiovascular disease, Intracranial aneurysm, Stroke, Subarachnoid hemorrhage

## Abstract

**Background:**

Rupture of an intracranial aneurysm resulting in a subarachnoid hemorrhage (SAH) is a life-threatening situation. Obesity is an increasing health challenge associated with numerous comorbidities. However, recent studies have shown a surprising decreased risk of SAH with increasing body mass index (BMI). The aim was to explore associations between other anthropometric variables and the rupture risk of an intracranial aneurysm, which to our knowledge is lacking in present literature.

**Methods:**

Using a bioelectrical impedance analysis device, we performed body composition analyses on 31 patients admitted with aneurysmal SAH (aSAH) and 28 patients with planned intervention on their unruptured aneurysm. We also collected information on comorbidities and relevant risk factors. Logistic regression was used to explore associations between anthropometric variables and patients with ruptured versus unruptured aneurysms.

**Results:**

Unadjusted estimates showed a significant inverse relationship between body fat percent and aneurysmal rupture (OR [95% CI]: 0.92 [0.86, 0.97], *P* = 0.009), and between body fat mass and aneurysmal rupture (OR [95% CI]: 0.95 [0.90, 0.99], *P* = 0.047). These risk relationships remained significant in age- and sex-adjusted analyses for body fat percent (OR [95% CI]: 0.93, [0.87, 0.97], *P* = 0.028), and body fat mass (OR [95% CI]: 0.95 [0.90, 0.99], *P* = 0.041).

**Conclusions:**

In recent studies showing a paradoxical relation between aSAH and obesity, BMI was the only parameter investigated. We further explored this “obesity paradox” and found lower body fat in aSAH patients compared to UIA. Future studies should investigate these relationships in larger samples.

*Clinical Trial Registration* NCT04613427, November 3, 2020, retrospectively registered

## Introduction

The rupture of an intracranial aneurysm (IA) leading to a subarachnoid hemorrhage (SAH) is a devastating incident causing death, or major consequences in the survivors. Intracranial aneurysms are relatively common, with a prevalence around 1–2% in Norway, and are most often asymptomatic [1]. The annual rupture risk of an unruptured intracranial aneurysm (UIA) is found to be around 1%, with some established risk factors, including age, hypertension, smoking, and female sex [2]. The risk of suffering an aneurysmal subarachnoid hemorrhage (aSAH) is found to be stable around 10 per 100.000 person years, although decreasing incidences are reported [3–5].

Obesity and associated comorbidities are causing increased morbidity and mortality worldwide [6–8]. Being overweight is a well-known risk factor for cardiovascular disease (CVD) and hypertension [9, 10], which increase the risk of aneurysm development and subsequently aSAH [11]. A recent systematic review found a significantly lower risk of aSAH in obese patients (body mass index (BMI) ≥ 30) compared to patients of normal weight (BMI < 25), but the studies were categorized as low quality [12]. Yet, studies investigating the impact of other anthropometric measures than BMI on the rupture risk of IAs are lacking. The aim of this study was therefore to explore associations between other anthropometric variables and the rupture risk of an intracranial aneurysm.

By using bioelectrical impedance analysis (BIA) technology, we measured body composition, such as body fat mass, body fat percent, visceral fat area, and fat-free mass, and explored the relationship with rupture risk of IAs. Although BIA is established in cardiovascular research, this method has, to the best of our knowledge, not been used before in the investigation of patients with IA.

## Materials and methods

### Study population

This study was approved in 2016 by the Regional Committee for Medical and Health Research (REK 2017/814) and performed according to the guidelines in the Declaration of Helsinki. It was conducted between April 2018 and June 2019 using a case–control design. Patients admitted acutely with aSAH and patients admitted electively with UIA for planned intervention or radiological control were included. All participants or next-of-kin signed a written informed consent form. We excluded patients with SAH caused by trauma or mycotic aneurysms, perimesencephalic SAHs, and UIA patients with a history of previous intracranial hemorrhage. Patients with a pacemaker or in pregnancy were excluded according to the BIA instructions.

Data on the patients’ age, gender, height, and medical history were collected from the patient and patient journals, while weight was measured by the department’s nurses. We defined aSAH as subarachnoid blood proven by computer tomography (CT) or lumbar puncture, and an aneurysm demonstrated with CT angiography, magnetic resonance angiography or conventional angiography. UIA was defined as one or multiple aneurysms confirmed by CT angiography or magnetic resonance angiography, admitted for radiological control or intervention.

Body composition was measured using a bioelectrical impedance analyser (InBody S10^®^; Biospace Co., Ltd., Seoul, Korea) with multiple frequencies (1 kHz, 5 kHz, 50 kHz, 250 kHz, 500 kHz, and 1000 kHz) and 8-point tactile electrodes. The electrodes were connected to the patients’ left and right thumb, middle finger, and ankles. All patients lied still for minimum 15 min to stabilize the body water distribution prior to the scan. Three measurements were recorded, and the mean value was calculated.

The data we collected from InBody S10 included visceral fat area, body fat mass, fat-free mass, skeletal muscle mass and calculation of percentage body fat.

The data that support the findings of this study are available from the corresponding author on reasonable request.

### Statistical analyses

Continuous variables are presented as mean (SD) and categorical variables as numbers (%). The statistical analyses were performed with R v3.6.1 (https://www.r-project.org), and data transformation and exploration were done by using the *tidyverse* packages (https://tidyverse.org).

Logistic regression analyses were used to obtain odds ratios (ORs) for associations between anthropometric variables and rupture risk (patients with ruptured versus unruptured aneurysms) using the *glm* function in the *stats* package v3.6.1 and the logit link function for the binominal coded outcome variable (0: UIA; 1: aSAH). The basic model (Model 1) was adjusted for age and sex. In model 2, we additionally included these established risk factors for aSAH: current smoking (no/yes), hypertension (no/yes), and family history of intracranial hemorrhage (no/yes). Statin use (no/yes) and alcoholism (no/yes) were also considered but not included due to total absence of cases in one or both groups. The extended model (Model 3) was further adjusted for CVD (no/yes). Here, diabetes (no/yes) and polycystic kidney disease (no/yes) were also considered but not included due to few or no cases.

## Results

**Fig. 1:**
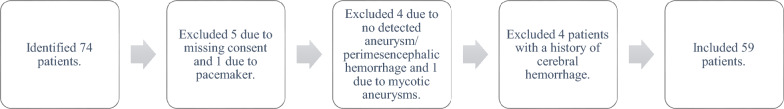
Flowchart of patient inclusion. Made by K. Løvik in Microsoft^®^ Word for Mac

From 74 initial patients, 15 were excluded according to the pre-specified criteria (Fig. 1). This resulted in a study population of 31 patients with aSAH and 28 patients with UIA. The patient characteristics are presented in Table 1.

**Table 1:** Characteristics of patients upon hospital admission.^1^

Variable	Ruptured aneurysm (31)	Unruptured aneurysm (28)	*P-*value^2^
Age	57 (SD)	60 (SD)	0.357
Female gender	17 (55%)	22 (79%)	0.099
Medical history			
Current smoker	12 (39%)	9 (32%)	0.501
Hypertension	12 (39%)	11 (39%)	0.963
Statin use	0	5 (18%)	0.055
Family history	2 (6%)	7 (25%)	0.118
Diabetes mellitus	1 (3%)	4 (14%)	0.309
PCKD	0	1 (4%)	0.972
CVD	6 (19%)	8 (29%)	0.649
Alcoholism	0	0	

*BMI* body mass index, *CVD* cardiovascular disease, *PCKD* polycystic kidney disease. Values are mean, or numbers (%). P-values were obtained from independent-samples t-test (age) or Chi-square tests (categorical variables). Made by K. Løvik in Microsoft^®^ Word for Mac

^1^Values are mean (SD) or numbers (%)

^2^P-values were obtained from independent-samples t-test (age) or Chi-square tests (categorical variables)

Unadjusted estimates showed a significant inverse relationship between body fat percent and aneurysmal rupture (OR [95% CI]: 0.92 [0.86, 0.97], *P* = 0.009), and between body fat mass and aneurysmal rupture (OR [95% CI]: 0.95 [0.90, 0.99], *P* = 0.047 (Table 2). These risk relationships remained significant in the age- and sex-adjusted analyses (Model 1) for body fat percent (OR [95% CI]: 0.93 [0.87, 0.99], *P* = 0.028) and body fat mass (OR [95% CI]: 0.95 [0.90, 0.99], *P* = 0.041). In this model, we also found an inverse, non-significant association between BMI and rupture risk (OR [95% CI]: 0.92 [0.83, 1.02], *P* = 0.138). When further controlling for current smoking status, hypertension, and family history of intracranial hemorrhage (Model 2), the associations were no longer significant for body fat percent (OR [95% CI: 0.93 [0.84, 1.01], *P* = 0.113) and body fat mass (OR [95% CI]: 0.93 [0.86, 1.00], *P* = 0.068). These and other risk relationships remained statistically non-significant when additionally adjusting for CVD (Model 3; Table 2).

**Table 2:** Ruptured aneurysms compared to unruptured IAs^1^

	Unadjusted estimates		Model 1		Model 2		Model 3	
	OR (95% CI)	*P*-value	OR (95% CI)	*P*-value	OR (95% CI)	*P*-value	OR (95% CI)	*P*-value
BMI (kg/m^2^)	0.95 (0.85, 1.04)	0.275	0.92 (0.82, 1.02)	0.138	0.90 (0.77, 1.03)	0.126	0.91 (0.79, 1.05)	0.210
Waist circumference (cm)	0.98 (0.94, 1.02)	0.371	0.98 (0.94, 1.02)	0.373	0.99 (0.93, 1.05)	0.690	0.99 (0.93, 1.05)	0.750
Visceral fat area (cm^2^)	1.00 (0.99, 1.01)	0.397	1.00 (1.00, 1.01)	0.518	1.01 (1.00, 1.02)	0.187	1.01 (1.00, 1.02)	0.163
Body fat mass (kg)	0.95 (0.90, 0.99)	0.047	0.95 (0.90, 0.99)	0.041	0.93 (0.86, 1.00)	0.068	0.94 (0.87, 1.01)	0.118
Body fat percent (%)	0.92 (0.86, 0.97)	0.009	0.93 (0.87, 0.97)	0.028	0.93 (0.84, 1.01)	0.113	0.93 (0.84, 1.02)	0.135
Fat-free mass (kg)	1.04 (1.00, 1.10)	0.070	1.02 (0.95, 1.09)	0.602	0.99 (0.91, 1.08)	0.838	1.01 (0.92, 1.11)	0.838
Skeletal muscle mass (kg)	1.06 (0.99, 1.15)	0.100	1.01 (0.91, 1.13)	0.858	0.96 (0.84, 1.09)	0.529	0.99 (0.86, 1.15)	0.901

*BMI* body mass index, *OR* odds ratio. Model 1: adjusting for age and sex. Model 2: additionally adjusted for current smoking, hypertension, and family history. Model 3: additionally adjusted for cardiovascular disease. Made by K. Løvik in Microsoft^®^ Word for Mac

^1^Model 1: adjusting for age and sex. Model 2: additionally adjusted for current smoking, hypertension, and family history. Model 3: additionally adjusted for cardiovascular disease

## Discussion

The present case–control study suggests a protective effect of increased body fat mass and body fat percent on the rupture risk of IAs. The significant inverse association is lost when adjusted for current smoking status, hypertension, and family history of ICH. A recent large study found that the previously reported inverse association between BMI and SAH in the literature appear to be explained by smoking and hypertension, thus, this may also be the case for our study [13]. Another large study found that increased BMI is significantly and inversely associated with saccular aneurysm rupture in males and patients aged ≥ 50 years, but the data is not adjusted for smoking and hypertension [14].

This is, to the best of our knowledge, the first case–control study on patients with ruptured compared to unruptured IAs exploring risk associations with anthropometric variables beyond BMI.

The pathogenesis of aneurysmal development and rupture is complex and not fully understood, and the inverse relationship with high body mass requires further investigations, but there are some possible explanations to discuss.

Inflammation is clearly associated with vessel wall degeneration, aneurysm formation and subsequently aneurysm rupture [15, 16]. Obesity is known to increase systemic inflammation, which should lead to an increased aneurysmal rupture risk [17]. However, studies have shown that an increased metabolic reserve of adipose could improve tolerance of catabolic and inflammatory events [12].

Body fat has shown to have surprising protective effects on various other diseases in clinical trials, for example on the prognosis with CVD [18]. In contrast, obesity also increases cardiovascular risk through risk factors such as dyslipidemia, but this is coincidentally associated with a lower risk of IA rupture [19, 20]. However, it is unclear whether the lower risk is caused by dyslipidemia itself or the use of statins, which provides a clear anti-inflammatory effect [20, 21]. These mechanisms may partly explain the inversed association between rupture risk and obesity.

Aneurysm formation is initiated by hemodynamically triggered endothelial dysfunction, which is an inflammatory response [22]. Body composition affects aneurysmal hemodynamics, such as vascular wall stress, which play an important role in the development and growth of IA [23]. A study on abdominal aortic aneurysms (AAA) have found higher peak wall stress in patients with low BMI compared to high BMI, and further, a higher rupture risk of AAA [24]. This may also be the case for intracranial aneurysms, as a recent study found that low aneurysmal wall shear stress (WSS) was associated with higher risk of IA rupture [25].

High body mass may increase central venous pressure and thereby an upstream effect of elevated intracranial pressure (ICP). Obesity has been linked to ICP pathogenesis in disorders such as idiopathic intracranial pressure (IIH) [26]. The association is poorly understood, but one potential pathway links metabolic disorders to elevated ICP through a thrombotic tendency due to dysregulation of haemostatic risk factors. Intracranial thromboses could subsequently cause impaired resorbtion of cerebrospinal fluid (CSF) and venous hypertension [27].

A suggested protective mechanism provided by high body mass is that an elevated ICP could counter the intravascular pressure, and thus, reduce the increased rupture risk that hypertension features on the aneurysm wall [28, 29].

Further, rapidly growing intracranial aneurysms have an increased rupture risk [30, 31].

It is possible that the counterpressure from an elevated ICP could prevent rapid aneurysmal growth and thereby reduce the rupture risk. In conclusion, the effect of obesity on ICP and aneurysmal growth is not fully understood and require further research in the future.

Most studies investigating the association between BMI and aSAH have focused on outcome and mortality. According to a recent systematic review, aSAH was associated with lower mortality in obese patients compared to normal-weight patients, but the studies were considered to be of low quality [12]. Some studies have related overweight to more favorable outcomes after aSAH [32, 33], while other studies report contradictory findings [34]. Feigin et al*.* found discordant effects of BMI on the risk of aSAH in a systematic review, with a longitudinal study showing a decreased risk of aSAH in patients with lean BMI and two case–control studies where lean BMI was associated with increased risk [11]. Sandvei et al*.* later found that overweight may be associated with reduced risk of aSAH [35]. Further larger case–control studies are required to conclude on the effect of BMI on the risk of aneurysmal rupture.

The present study is limited by our recruitment strategy to include all patients admitted with UIA and aSAH, which means we could not have matching controls. Inclusion of sudden death aSAHs was not practically possible, and could provide a selection bias, but this is a common practice in similar studies. The low sample size in this study is a strongly limiting factor, which affects the impact of the study. Our initial calculations based on previous years of admission numbers would provide a total patient number of 100 (70% aSAH) in a 1-year period. During the last 10 years, the number of patients admitted with a subarachnoid hemorrhage has decreased worldwide, also in Norway, which may partially be explained by the significant decrease in smoking in the population [36, 37].

Another limiting factor is that anthropometric data was measured after 2–3 days after admission, on average. As the patients suffering from aSAH are prone to more bed rest than patients treated electively for UIA, the measured effect of lower body fat in aSAH patients could partly be explained by bed rest. However, according to studies, unlike muscle mass, body fat mass is not severely affected by bed rest [38, 39].

Although measurements of body composition were performed by the same person using the mean value of three reliable measurements after the suggested 15 min bed rest, the technique has limitations due to human error.

Our study has several strengths. All patients were included from the same catchment area and the same hospital, giving the opportunity to study rupture risk in a well-defined population, and with the same classification of characteristics. We had access to individual patient data, which made it possible to conduct multivariate analyses. The exclusion of previous intracranial hemorrhage reduced possible selection bias and misclassification bias.

Body composition analyzers, e.g., different versions of BIA technology, such as InBody^®^, have been utilized in the last decade in clinical research, including on cardiovascular disease [40, 41]. The validity of BIA has been compared to Dual-Energy X-ray Absorptiometry (DEXA) and found equally strong in multiple studies [42, 43]. To the best of our knowledge, BIA has never before been used in a neurosurgical setting.

## Conclusion

The current study indicates that body fat mass percent were inversely associated with rupture risk of IAs when adjusted for age and sex, but not when adjusted for further parameters such as smoking and hypertension. The clinical implications should obviously be discussed with caution but could prove to be important when consulting patients with UIA and whether to choose treatment or expectance. This study may implicate that one may consider a more expecting treatment plan towards patients with high body fat percent.

## Data Availability

Available at reasonable request.

## Abbreviations

AAA: Abdominal aortic aneurysm
aSAH: Aneurysmal subarachnoid hemorrhage
BIA: Bioelectrical impedance analysis
BMI: Body mass index
CI: Confidence interval
CVD: Cardiovascular disease
CVP: Central venous pressure
IA: Intracranial aneurysm
ICP: Intracranial pressure
IIH: Idiopathic intracranial hypertension
OR: Odds ratio
PCKD: Polycystic kidney disease
SAH: Subarachnoid hemorrhage
UIA: Unruptured intracranial aneurysm
